# First DNA Barcoding Survey in Bulgaria Unveiled Huge Diversity of Yeasts in Insects

**DOI:** 10.3390/insects15080566

**Published:** 2024-07-26

**Authors:** Roumen Dimitrov, Dilnora Gouliamova, Borislav Guéorguiev, Maudy Smith, Marizeth Groenewald, Teun Boekhout

**Affiliations:** 1 Institute of Mathematics and Informatics, Bulgarian Academy of Sciences, G. Bonchev 8, 1113 Sofia, Bulgaria; 2 The Stephan Angeloff Institute of Microbiology, Bulgarian Academy of Sciences, G. Bonchev 26, 1113 Sofia, Bulgaria; 3 National Museum of Natural History, Bulgarian Academy of Sciences, bul. “Tsar Osvoboditel” 1, 1000 Sofia, Bulgaria; gueorguiev@nmnhs.com; 4 Westerdijk Fungal Biodiversity Institute, Uppsalalaan 8, 3584 CT Utrecht, The Netherlands; m.smith@westerdijkinstitute.nl (M.S.); m.groenewald@wi.knaw.nl (M.G.); 5 The Yeast Foundation, 1015 JR Amsterdam, The Netherlands; teun.boekhout@gmail.com; 6 College of Sciences, King Saud University, Riyadh 11451, Saudi Arabia

**Keywords:** biodiversity survey, taxonomy, insects, yeasts

## Abstract

**Simple Summary:**

This study surveyed the diversity of yeast species in the guts of various insects from three Bulgarian National Parks, Vitosha, Rila, and Pirin. Insects from a wide range of taxonomic groups, including Coleoptera, Orthoptera, Lepidoptera, Hymenoptera, Dermaptera, and Collembola, were collected. Yeast strains were identified using DNA barcoding of ribosomal markers. This study found 89 ascomycetous and 18 basidiomycetous yeast isolates. Forty-two percent of the yeast isolates represented novel yeast species. Our study confirms that insects remain a rich source of unknown yeast species.

**Abstract:**

In this study, we conducted a comprehensive survey aimed at assessing the diversity of yeast species inhabiting the guts of various insect species collected mainly from two Bulgarian National Parks, namely, Rila, and Pirin. The insect specimens encompass a broad taxonomic spectrum, including representatives from Coleoptera, Orthoptera, Lepidoptera, Hymenoptera, Dermaptera, Isopoda, and Collembola. Yeast strains were identified with DNA barcoding using the ribosomal markers, specifically, the D1/D2 domains of the ribosomal large subunit (LSU) and the internal transcribed spacers regions ITS 1 + 2 (ITS). The analysis unveiled the presence of 89 ascomycetous and 18 basidiomycetous yeast isolates associated with the insect specimens. Furthermore, our study identified 18 hitherto unknown yeast species.

## 1. Introduction

In various regions around the world, including Bulgaria, environmental and climate changes have led to a rapid decline in biodiversity [1]. This critical situation emphasizes the necessity for conducting comprehensive biodiversity research and conservation efforts. Yeasts have ecological, medical, and biotechnological significance, inhabiting various natural ecosystems, such as soil, water, plants, and animals [2,3]. Insects, constituting the largest phylum by species diversity with an estimated 5.5 million species, stand out among animal taxa as a well-known but not well explored habitat for yeasts [4]. In forest ecosystems, insects play a pivotal role in nutrient cycling, pollination, and plant seed dispersal. Yeasts and insects engage in various symbiotic interactions. This includes mutualism, wherein yeasts contribute to nutrient breakdown through enzymatic processes [5], furnish insects with nutrients and essential vitamins, and provide antimicrobial defense [6,7,8]. In exchange, insects offer a secure niche for yeast reproduction, act as transportation agents, and facilitate access to food sources in natural environments [6,9]. Despite the well-documented importance of these associations, our knowledge of yeast biodiversity associated with insects in various regions of our planet remains limited. Most studies on yeast biodiversity have been concentrated in regions such as Western Europe, Japan, and North America [10,11]. Moreover, most yeast strains maintained in world culture collections were isolated from food products, plants, and human sources [3].

Studies on yeasts associated with insects have revealed the presence of many unknown species in insect guts [12,13,14,15,16,17,18,19,20,21,22]. It has been suggested that further exploration of insects in less-studied regions may lead to the discovery of additional novelties [11]. For instance, recent publications [23,24] have unveiled a significant diversity of non-*Saccharomyces* yeast species associated with dung beetles collected from Botswana, a scarcely studied region in Africa.

Bulgaria, one of Europe’s most biologically diverse countries, owes its rich biodiversity to a varied climate and geography. It is located in the eastern part of the Balkan Peninsula and belongs to the Holarctic floristic region. The country’s complex geological history, mountains with highly dissected topography, river valleys, and basin fields, as well as the influence of marine basins in the east and south, contribute to a diverse climate that creates conditions for varied vegetation and rich flora. Approximately 35% of Bulgaria’s land area is covered by forests, housing around 4030 vascular plant species [25], including 172 endemics [26]. The country also hosts over 4870 known fungal species [27], along with 20,900 species of insects [28]. This wealth of flora and fauna provides potential habitats for numerous yet undiscovered yeast species with possible important biotechnological properties.

The systematics of yeasts has evolved alongside major advancements in biological science. Initially, microscopy facilitated yeast species identification through the examination of phenotypic markers, including cell and colony morphology. However, these markers often lack resolution due to the extensive biodiversity of yeast. Advances in biochemistry enabled the utilization of biochemical characteristics such as the assimilation and fermentation of various carbohydrates, as well as the assimilation of nitrogen compounds. Progress in molecular biology has enabled the use of proteins, DNA, and RNA as markers. In 1951, the complete amino acid sequence of the insulin molecule was determined [29]. For the first time, it was shown that every protein has a unique sequence with 20 different amino acids at each position. The number of protein characteristics, for comparison, equals to 20^N^ (20 amino acids, N length of the protein). In 1962, based on measurements of the rate of mutations in hemoglobin molecules of closely related species, the concept of a molecular clock was introduced [30]. This concept is based on the idea that mutations accumulate at a fairly consistent rate, serving as a “clock” that can be used to estimate the time since two species shared a common ancestor. From then on, it became possible to calculate the evolutionary distance between organisms and construct phylogenetic trees based on molecular sequences. In 1977, biology was revolutionized by the proposition of ribosomal RNA (rRNA) as a universal barcoding marker for the identification of organisms and the determination of genetic relationships among them [31]. The rRNA marker has superior discriminative power compared to proteins; for example, in the case of 18S rRNA, the number of characteristics is equal to 4 ^1789^ (4 different nucleotides, 1789 the length of *Saccharomyces cerevisiae* 18S rRNA).

Pioneering studies by Cletus Kurtzman on the sequence analysis of the D1/D2 region of the LSU (~600 nucleotides) of 500 known yeast species demonstrated that conspecific yeast strains may exhibit up to three nucleotide differences, and a separate species may differ by six or more nucleotides [32,33]. This approach was also applied to the delimitation of basidiomycetous yeast species by Jack Fell [34]. Along with the LSU marker, the ITS region is widely used for yeast identification [35,36,37,38,39,40]. The LSU and ITS ribosomal markers are effective for the identification of most yeasts, but there are cases when they were found to be unable to discriminate closely related yeast species. To overcome this, multigene analysis using additional protein-coding gene sequences has been suggested [41,42]. Sequence identity cut-off values for the LSU and ITS markers were determined based on the analysis of a large dataset, including 9000 CBS yeast strains belonging to 2000 known yeast species [43].

The present study aims to contribute to our understanding of yeast biodiversity by focusing on the yeasts associated with insects collected in Bulgaria. We used combined sequence analyses of the ITS and LSU ribosomal DNA markers for the identification of the isolated yeast strains [37,38,39,40].

## 2. Materials and Methods

### 2.1. Insect Collection and Isolation of Yeasts

The collection of insects was conducted utilizing entomological methodologies involving searches conducted beneath stones, logs, and tree bark, as well as on mushrooms and within decaying wood. Furthermore, a thorough entomological examination of plant leaves and flowers was undertaken. The insect sampling encompassed a wide spectrum of plant communities, including xerophilic, deciduous, mixed, and coniferous forests, as well as wetlands, arid grasslands characterized by herbaceous vegetation, and open riparian shrub and forest ecosystems. The altitudinal gradient spanned from 70 to 1600 m above sea level.

To prevent any potential cross-contamination, each individual insect specimen was transported within separate sterile containers from the respective collection sites to the laboratory. Following the initial taxonomic identification, which took place at the National Museum of Natural History in Sofia, those insects designated for yeast isolation purposes were subsequently transported to the Institute of Microbiology, Bulgarian Academy of Sciences, located in Sofia. The insects were stored at −80 °C. Prior to the dissection process, the insects underwent a thorough cleansing procedure involving a wash with 70% ethanol, followed by rinsing with 0.7% sterile saline solution. As a quality control measure, the saline wash was cultured on an acidified yeast extract–malt extract agar (YM agar, pH 4.0–4.2) plate, serving as a negative control. To aseptically extract the gut contents from the insect bodies, the dissection procedure was carried out under a microscope, utilizing a sterile Petri dish. Subsequently, the gut contents were gently crushed within a sterile Eppendorf tube containing 0.7% sterile saline. The crushed gut contents were then plated on acidified YM agar plates (pH-3.4) and incubated at ambient room temperature. Following an incubation period of three days, colonies displaying a yeast-like morphology were selectively isolated and sub-cultured as necessary until pure yeast cultures were obtained, following established protocols [44].

### 2.2. Yeast Identification

Morphological, biochemical, and physiological characterization of the isolates was performed according to established methodsls [44,45,46,47].

### 2.3. Yeast Strain Deposit in Culture Collections

New yeast strains obtained in this study were deposited in the CBS culture collection of the Westerdijk Fungal Biodiversity Institute (CBS), Utrecht, The Netherlands, and the yeast culture collection of the National Bank for Industrial Microorganisms and Cell Cultures (NBIMCC), Sofia, Bulgaria.

### 2.4. DNA Barcoding Analysis

The LSU and ITS regions were amplified directly from individual yeast colonies without extracting DNA from cells [14]. The primer sets V9G and LS266 were used for the amplification of the LSU and ITS sequences [48]. A cell suspension of one loopful of cells in 50 μL of autoclaved water was heated at 95 °C for 5 min, and 2 μL of the supernatant was used as a template in 25 μL PCR mixture containing 7.5 μL of PCR master mix (Fermentas, Darmstadt, Germany), 0.5 μM of each primer. The amplified products were purified using a QIAquick purification kit (QiaGen, Germantown, MD, USA) according to the manufacturer’s instructions. Direct sequencing of the LSU and ITS regions was performed using primers NL1/NL4 [49] and ITS1/ITS4 [50], respectively, with a Taq DyeDeoxy terminator cycle sequencing kit (Applied Biosystems, Foster City, CA, USA) by Macrogen Inc. (Seoul, Republic of Korea), according to the manufacturer’s protocol. Purified sequencing reaction mixtures were separated with a 3730XL automated DNA analyzer (Applied Biosystems, Foster City, Foster City, CA, USA). The program BLAST from NCBI (https://www.ncbi.nlm.nih.gov, accessed on 1 July 2024) was used to compare the sequences to known LSU and ITS sequences for the assignment of the closest related taxa [51]. All the LSU and ITS sequences from this study were submitted to the GenBank database under the accession numbers listed in Tables S1 and S2 (Supplementary Materials).

## 3. Results and Discussion

### 3.1. Insect Collection and Distribution of Yeasts across Insects Orders

**Figure 1:**
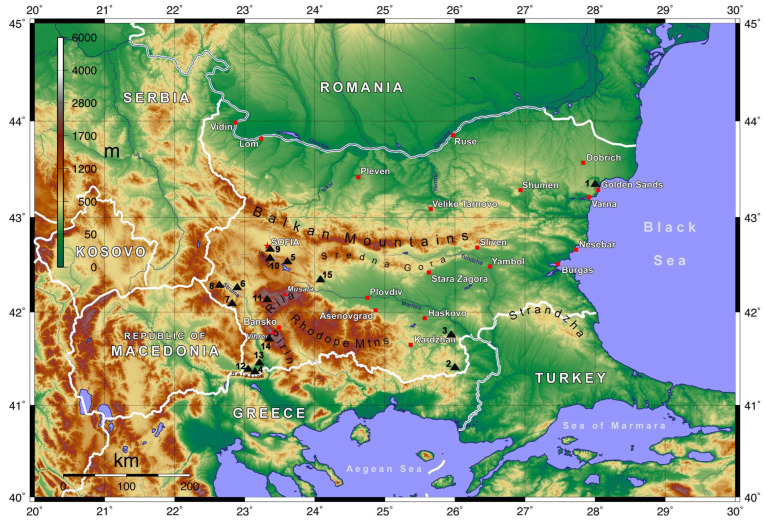
Insect collection sites. The numbers 1–15 correspond to the entries in column 1 of Table 1. Red squares denote major settlements, the red pentagram indicates the capital city, and red triangles highlight the country’s highest peaks.

Insects were collected from diverse regions across Bulgaria during the following three periods: May 2008, April to September 2009, and July to September 2012 (Figure 1). Additional pertinent details concerning the precise locations, altitudes, and seasons of collection are outlined in Table 1.

More than 170 insect specimens were collected. They belonged to various insect orders, including Coleoptera, Orthoptera, Lepidoptera, Hymenoptera, Dermaptera, and Collembola. The isolation of yeast strains from the insect guts yielded a total of 107 strains, distributed across 28 insect families. The breakdown is as follows: 14% from longhorn beetles (Cerambycidae), 13% from unidentified Coleoptera spp., 10% from darkling beetles (Tenebrionidae), 8% from ground beetles Carabidae, 7% from scarab beetles (Scarabaeidae), 6% from Cetoniidae, 5% from Orthoptera. 3.7% from Hymenoptera, 3.7% from leaf beetles (Chrysomelidae), 2.8% from Ciidae, and 2% each from Staphylinidae, Ruthelidae, Geotrupidae, Buprestidae, Melandryidae, Dermaptera, Lepidoptera, and Hexapoda. The twelve remaining insects collectively contributed 10.8%.

In most instances, each individual insect specimen was found to harbor a single yeast species. However, it is worth noting that there were exceptions, with certain insects containing as many as three distinct yeast species.

*Bolitophagus reticulatus* (Tenebrionoidea) found in National park Rila contained two species, while *Bolitophagus interruptus* found in the Podgorie area contained three species; two species were isolated from *Cetonia aurata* (Cetoniidae) found in East Rhodopes, two species were recovered from Sminthuridae sp. (Collembola) found on a fungus in Nature park Vitosha, as well as from an unknown Orthoptera spp. collected from a plant on the bank of the Struma River (Supplementary Materials, Table S1).

### 3.2. Ascomycetous Yeast Associated with Insects

DNA barcoding analysis revealed that 89 isolates of yeasts were affiliated with ascomycetous yeast genera—*Candida*, *Debaryomyces*, *Diutina, Exophiala*, *Hanseniaspora*, *Kazachstania*, *Kluyveromyces*, *Lachanceae*, *Metschnikowia*, *Meyerozyma*, *Nakaseomyces*, *Ogataea*, *Pichia*, *Priceomyces*, *Saccharomyces*, *Scheffersomyces*, *Starmerella*, *Wickerhamiella* and *Wickerhamomyces*.

Genus *Scheffersomyces* Kurtzman and M. Suzuki (2010). Seventeen yeast isolates of the genus *Scheffersomyces* were obtained from various insects. Ten isolates were obtained from Cerambycidae beetles, while two isolates came from beetles of the Lucanidae and Tenebronidae families and an unidentified Zygaenidae moth (Supplementary Materials, Table S1). Four isolates originated from an unidentified Coleoptera spp. and the larva of *Cetoniidae* sp. Five yeast strains were identified as *Scheffersomyces stipitis.* Twelve strains belonged to *Scheffersomyces insectosa*. *Scheffersomyces* yeasts have been isolated from wood-infesting insect larvae and their frass, from the guts of lignicolous beetles, and from rotten wood in Europe and North and Central America [52]. Members of the genus *Scheffersomyces, Sch. stipitis*, and *Sch. shehatae* are of particular interest due to their ability for the fermentative production of ethanol from D-xylose, a major pentose found in plant biomass [53]. A biodiversity survey conducted in the USA and Panama showed the consistent presence of *Scheffersomyces* yeasts in the gut of *Odontotaenius disjunctus* (Passalidae) [54,55]. A symbiotic relationship was proposed, wherein the yeast *Sch. stipitis* metabolizes D-xylose to the advantage of the associated insects [54]. Another survey of yeast associated with passalids in Guatemala and Thailand showed that *Scheffersomyces* yeast was the most abundant species in the gut of both wood roaches and Guatemalan passalids, while in Thai passalids, the most abundant yeasts were closely related to *Candida insectamans* [56]. *Scheffersomyces* yeasts were also shown to occur in the gut of the North American wood roach *Cryptocercus* spp. (Blattodea: Cryptocercidae) [57].

Genus *Suhomyces* M. Blackwell and Kurtzman (2016). Five strains of *Suhomyces atakaporum* were found in the gut of beetles belonging to various families, including Tenebrionidae, Cerambycidae, Erotylidae, and Melandryidae (Supplementary Materials, Table S1). *Suhomyces tanzawaensis* was isolated from mosses in Japan [58], and it had no known close relatives for a long time. An additional five species were isolated from soil, rotten wood, mushroom fruiting bodies, frass of moss larva, and a Bostrichidae beetle collected in the USA, Australia, and South Africa [59]. Sixteen new species, including *Su. atakaporum,* were discovered from fungus-feeding beetles collected in Panama and the USA [14]. The species form a well-supported monophyletic clade and were isolated from 11 Coleoptera families, with the majority coming from Tenebrionidae and Erotylidae beetles. A yeast biodiversity survey conducted in Thailand showed that almost half of the 128 collected Thai strains were members of the *Su. tanzawaensis* clade. Three species, *Suhomyces panamericana, Suhomyces anneliseae,* and *Suhomyces chickasaworum*, common in the Western Hemisphere, also occurred in Thailand [56].

Genus *Debaryomyces* Lodder and Kreger-van Rij (1952): Two yeast isolates obtained from the beetle species *Lachnaia sexpunctata* (Chrysomelidae) and an unidentified grasshopper (Orthoptera) (Table S1) were identified as *Debaryomyces fabryi*. It has been demonstrated that ribosomal markers are insufficient for distinguishing species of *Debaryomyces hansenii, D. fabryi*, and *Debaryomyces subglobosus* [42]. Therefore, for accurate identification of these isolates, analysis of an additional marker, the actin gene (ACT1), is necessary. [42]. Previous studies reported on the isolation of *Debaryomyces* yeasts from Central European bumblebees [60] and the beetle species *Melanotus villosus* (Elateridae) collected in the UK [61]. These data highlight the coexistence of *Debaryomyces* species within different host organisms.

Genus *Schwanniomyces* Klöcker (1909): Two strains of *Schwanniomyces vanrijiae* were isolated, one from the larva of *Cetonia aurata* (Cetoniidae) and the other from the gut of *Harpalus anxius* (Carabidae). *Schwanniomyces vanrijjiae* and *Schwanniomyces polymorphus* were shown to be associated with wood ants, *Formica aquilonia* [62], and carpenter ants, *Camponotus vicinus* [Formicidae] [63]. Experiments conducted on *Camponotus vicinus* larvae, which were fed a nutrient-deficient diet and exposed to live yeast *Schw. polymorphus*, demonstrated that the yeasts increased brood weight and facilitated the complete development of larvae into adult ants. This led to the conclusion that yeasts provide ants with essential substances for their nutrition and development [64].

Genus *Metschnikowia* Kamienski (1899). Two strains of *Metschnikowia pulcherrima* were isolated from an unidentified butterfly species (Lepidoptera) and a bumblebee (*Bombus* sp.: Apidae). A strain of *Metschnikowia reukaufii* was isolated from a *Buprestidae* spp. *Metschnikowia* yeasts were consistently isolated from the gut of diverse insects. Woolfolk and Inglis isolated *Metschnikowia pulcherrima* from *Chrysoperla rufilabris* (Chrysopidae) populations in the USA [65]. Three novel *Metschnikowia* species were isolated from *Chrysoperla* species collected in Arizona [66]. *Metschnikowia chrysoperlae* was found in the gut or frass of corn-feeding caterpillars *Ostrinia nubilalis* (Crambidae) in Austria, as well as in corn earworm *Helicoverpa armigera* (Noctuidae) and corn rootworm *Diabrotica virgifera* (Chrysomelidae) [67]. *Metschnikowia* yeasts were also found in the gut of lacewings, soldier beetles, and leaf beetles in the United States and Panama [20]. A survey of yeasts associated with social wasps in the USA has shown that about 25% of identified yeast belonged to the genus *Metschnikowia* [68]. Recently, a new species *Metschnikowia baotianmanensis* was shown to be associated with a rhinoceros beetle, i.e., *Allomyrina dichotoma* (Scarabeidae) collected in China [69].

Genus *Hypopichia* von Arx and van der Walt (1976): In the genus *Hypopichia*, two strains of *Hypopichia rhagii* were isolated from *Dinoptera colaris* (Cerambycidae) and a larva of Buprestidae sp. In Europe, *H. rhagii* was solated from both the adult and larval stages of *Rhagium inquisitor* (Cerambycidae) and the species was considered a symbiont of this insect [70]. Subsequent studies on gut symbionts of Cerambycidae beetles in Germany [22] further confirmed the association of *H. rhagii* with *R. inquisitor*. Our findings expand this association to include additional beetle species.

Genus *Saccharomyces* Meyen ex Reess (1870). In the present study, one strain was identified as *Saccharomyces cerevisiae* and was isolated from the beetle *Mimela aurata* (Rutelidae) (Supplementary Materials, Table S1). The correct identification of this isolate requires the analysis of several additional markers, namely, translation elongation factor 1-α (EF1-α), mitochondrial small-subunit rDNA, and cytochrome oxidase II (COX II), because the LSU and ITS markers alone could not distinguish closely related species, namely *S. cerevisiae, S. paradoxus*, and *S. cariocanus* [41].

*Saccharomyces* yeasts were found in the gut of Corydalidae (Megaloptera) [21] and are well-known to be associated with *Drosophila* flies [71,72]. A yeast survey conducted in Europe demonstrated that social wasps (Apocrita: Hymenoptera) serve as a reservoir and as a dispersion vector of *S. cerevisiae* in nature [73]. The yeast was also present in the gut of rhinoceros beetles (Scarabaeidae) [69].

Genus *Lachancea* Kurtzman (2003). In this study, a strain of *Lachancea kluyveri* was isolated from the beetle *Purpuricenus budensis* (Cerambycidae). *Lachancea thermotolerans*, and *L. fermentati* were frequently isolated from the gut of dobsonfly (Megaloptera: Corydalidae) [21]. A study of yeasts associated with social wasps conducted in the USA revealed that approximately 30% of yeast isolates belonged to *L. kluyveri*, *L. thermotolerans*, and *L. fermentati* [68]. This finding emphasizes the importance of *Lachancea* species in diverse insects, encompassing beetles, dobsonflies, and social wasps.

Genus *Hanseniaspora* Zikes (1912). Two strains of *Hanseniaspora uvarum* were isolated, one from an unidentified Coleoptera spp. and the other from *Forficula auricularia* (Dermaptera: Forficulidae) found on a vine tree. In prior research, *Hanseniaspora viniae* yeasts were isolated from the surface and the gut of Corydalidae (Megaloptera) and Ascalaphidae (Neroptera) [21]. In another survey, *Hanseniaspora* was the most dominant yeast in *Drosophila* species in decaying fruits [72]. Interestingly, the yeast was not found in *Drosophila* flies that feed on flowers, suggesting that the diet of *Drosophila* shapes its yeast symbiont community. In another study, *Hanseniaspora* species were the most frequently isolated yeast from the fruit fly *Bactrocera tryoni* (Diptera: Tephritidae) [74]. Experimental data demonstrated that *H. uvarum* plays a beneficial role in the survival and fitness of the fly larvae.

Genus *Starmerella* Rosa and Lachance (1998). Two species, *Starmerella bombicola* and *Starmerella bombi*, were isolated from the beetle *Valgus hemipterus* (Scarabaeidae) and from *Bombus* sp., correspondingly (Table S1). *Starmerella* yeasts have predominantly been obtained from habitats in tropical and temperate regions of the Western Hemisphere, as well as the Australia-Pacific biogeographical zone. They exhibit an ecological association with flowers, along with their attendant bees and wasps, as well as beetle species belonging to the families Nitidulidae and Chrysomelidae [75].

Species belonging to an unaffiliated clade (Table S1). Three strains of *Candida savonica* were consistently isolated from *Ciidae* spp. found on the fungus *Trametes versicolor* (Polyporaceae) in two different locations (Table S1). Based on our observations, we suggest the possibility of an association between *C. savonica* and *Ciidae* spp. Single isolates of *C. schatavii* and *C. boleticola* were obtained from an unidentified grasshopper (Orthoptera) and the bracket fungus *Ganoderma* spp. (Ganodermataceae), respectively.

Opportunistic Yeast Pathogens: We obtained an isolate of the opportunistic pathogen *Nakaseomyces glabratus* (formerly *Candida glabrata*) from *Oxythyrea funesta* (Cetoniidae). *Nakaseomyces glabratus* is ranked as the second most prevalent causative agent of candidiasis globally [76]. Isolates of *Candida maltosa* and *Candida zeylanoides* originated from the gut of the beetles *Rhaesus serricollis* (Cerambycidae) and *Cybister lateralimarginalis* (Dytiscidae) (Table S1, Supplementary Materials).

Additionally, an isolate of the opportunistic human pathogen *Meyerozyma guilliermondii* was found in the gut of the beetle *Bitoma crenata* (Colydiidae). This yeast has been isolated from various beetles, including Cerambycidae [77], Scarabaeidae [78], Curculionidae [79], and Chrysomelidae. It has also been found in grass moss (Lepidoptera: Crambidae) [80], ants (Formicidae) [81], mining bees (Hymenoptera: Andrenidae), a mushroom-feeding fly [82], dobsonflies (Corydalidae: Megaloptera), and owlflies (Ascalaphidae: Neroptera) [19]. Recent studies have demonstrated that *Me. guilliermondii* was the third most frequently isolated species from the gut of dung beetles (Scarabaeidae) collected in Botswana [23]. Notably, *Me. guilliermondii* is the sixth most frequently isolated yeast in clinical environments, as reported by Pfaller et al. [83].

Opportunistic pathogens, including *Candida orthopsilosis*, *C. maltosa*, *Candida parapsilosis*, *Candida tropicalis*, *Candida neerlandica*, and *Lodderomyces elongisporus*, were found in the guts of different insects in the USA and Panama [19]. A study conducted in Brazil demonstrated that leaf-cutting ants (Formicidae: Attini) harbored the yeast *C. parapsilosis* [84]. These findings highlight the significant role of insects as major vectors for the dispersal of potential pathogenic yeasts in natural ecosystems.

Other yeast species isolated in this study: Five yeast strains belonged to the species *Exophiala cideris* (black yeast), *Kluyveromyces dobzhanskii, Pichia membranifaciens, Teunomyces kruisii,* and *Yarrowia lipolitica.* They were isolated from various insects as presented in Table S1.

### 3.3. Novel Ascomycetous Yeast Species

A DNA barcoding analysis revealed several novel species, some of which have already been validly described in our previous articles, namely, *Ogatae saltuana* (Curculionidae: Scolitinae sp.) [85], *Priceomyces vitoshaensis* (Carabidae: *Pterostichus melas*) [86], *Nematodospora valgi* (Scarabaeidae: *Valgus hemipterus*), and *Candida cetonii* (Cetoniidae: *Oxythyrea funesta*) [37], *Yarrowia parophonii* (Carabidae: *Paraphonus hirsutulus* and Coleoptera spp.) [87], *Kazachstania molopis* (Carabidae: *Molops piceus*) [88], *Kazachstania chrysolinae* (Chrysomelidae: *Chrysolina polita*) [38,39], *Suhomyces rilaensis* (Tenebrionidae: *Bolitophagus interruptus*; *B. reticulatus*) [89], and *Starmerella xylocopis* (Hymenoptera: Aphidae) [90].

Two isolates, 91W and 91WR (100% sequence similarity in both the LSU and ITS), originated from the beetle *Carabus violaceus azurescens* (Carabidae; Supplementary Materials, Table S1). A blast analysis showed that the most similar sequences in the GenBank database belong to *Starmerella sorbosivorans* CBS 8768^T^ with 93% identity in the LSU (28 nucl. subst., 1 gap) and *Candida spenceri* CBS 11673^T^, with 87% identity in the ITS (27 nucl. subst., 20 gaps).

Two isolates, DZ1_1 and DZ2_1, came from the beetle *Bolitophagus interruptus* (Tenebrionidae) found on *Ganoderma* spp. (Ganodermataceae). An additional isolate, D300, was obtained from the specimen of *Bolitophagus reticulatus* (Tenebrionidae) found on the fungus *Fomes* spp. (Polyporaceae). A pairwise sequence analysis showed that the strains have 86% sequence identity (51 nucleotide substitutions and 24 gaps) in the LSU with *Wickerhamiella musiphila* CBS 10697^T^ and 84% identity (62 nucleotide substitutions, 20 gaps) in ITS sequences with *W. infanticola* CBS 11940^T^.

Additionally, two isolates, DZ1_2 and DZ2_2 (100% sequence similarity in both the LSU and ITS), were isolated from the beetle species *Bolitophagus reticulatus* (Tenebrionidae; Table S1). The analysis showed that the strains show 99% identity (three nucl. subst. in the LSU) and 93% identity (23 nucl. subst., 12 gaps in the ITS) with *Blastobotrys peoriensis* CBS 10340^T^.

The analysis showed that nine isolates, IMB-YP, IMB-P1, IMB-PP2, IMB-P21, IMB-P5, IMB-P72, IMB-YP8, IMB-PX1, and IMB-PG2, showed 100% sequence similarity in the LSU and 99–100% sequence similarity in the ITS. The strain showed 100% sequence similarity in the LSU and 86% sequence similarity in the ITS (41 nucl. subst., 14 gaps) of the new strain IMBP43, 90% sequence similarity in the LSU of *Suhomyces panamericana* ATCC MYA-4318^T^ (45 nucl. subst., 11 gaps), and 84% identity in the ITS of *S. tanzawaensis* CBS 7422^T^ (46 nucl. subst., 22 gaps).

The isolate IMB-P43 showed 100% sequence similarity in the LSU and 86% sequence similarity in the ITS (41 nucl. subst., 14 gaps) of the nine strains, IMB-YP, IMB-P1, IMB-PP2, IMB-P21, IMB-P5, IMB-P72, IMB-YP8, IMB-PX1, and IMB-PG2, 94% similarity (19 nucl. subst., five gaps in the LSU) with *Suhomyces ambrosiae* CBS 8844^T^, and 91% similarity (21 nucl. subst., seven gaps in the ITS) with *Suhomyces chikasaworum* CBS 9830^T^.

A blast analysis showed that two isolates, D15 and D24 (100% sequence identity in both the LSU and ITS), have 88% sequence identity (53 nucl. subst., 11 gaps in the LSU and 52 nucl. subst., 23 gaps in the ITS) with *Diutina ranongensis* CBS 10861^T^.

### 3.4. Basidiomycetous Yeast Associated with Insects

The LSU and ITS sequences of 18 yeast isolates showed similarities with that of members of basidiomycetous yeast genera as retrieved from the GenBank database.

Genus *Filobasidium* L.S. Olive (1968). The most frequently isolated yeast species belonged to the genus *Filobasidium*, with the four species *Filobasidium stepposus, Filobasidium oeirensis, Filobasidium chernovii,* and *Filobasidium wieringae.* These yeasts were isolated from beetles belonging to the families Chrysomelidae, Silvanidae, Buprestidae, and Cerambycidae (Supplementary Materials, Table S2).

Genus *Apiotrichum* Stautz (1931). Three strains belonged to two species of the genus *Apiotrichum*, namely, *Apiotrichum lignicola* and *Apiotrichum humicola*, and originated from beetles of the families Geotrupidae and Staphylinidae.

Additional yeast species isolated in this study: *Naganishia friedmanii, Rhodosporidium kratochvilovae, Vanrija albida, Rhodotorula mucilaginosa,* and *Trichosporon coremiiforme*. They originated from the gut of insects belonging to Cerambycidae, an unidentified grasshopper (Orthoptera sp.), and Alexiidae sp. (Table S2).

Prior to our research, species from the genus *Vanrija*, i.e., *V. humicola* and *V. musci*, and from the genus *Trichosporon*, i.e., *T. dermatis, T. moniliiforme,* and *T. porosum*, were found in Guatemalan and Thai passalids [56]. *Trichosporon* species were identified in insect frass from Thailand [91] and in the guts of beetles (*Scarabaeidae,* Tenebrionidae) collected across diverse locations, including Panama, South Africa, and the USA [92,93,94]. The species from the genera *Apiotrichum, Naganishia, Rhodotorula*, and *Trichosporon* were also found in the guts of dung beetles collected in Botswana [23].

*Cystobasidium psychroaquaticum* [95], was isolated from an unidentified springtail (Collembola: Sminthuridae).

Four yeast isolates, D54, D59-1, D55, and D60-1, exhibited 99% sequence identity in the LSU (nine nucl. substit.) of *Trichosporon asteroides* CBS 6183 and 99% identity in ITS (four nucl. subst., two gaps) of *T. faecalis* CBS 4228T.

A single isolate, D162_2, originating from a Melandrydae beetle spp. showed 92% sequence similarity with the LSU (47 nucl. subst., 5 gaps) of *Saitozyma flava* CBS 331^T^ and 89% similarity with ITS (15 nucleotide substitutions, 12 gaps) of *Teunia cuniculi* CBS 10309^T^ and represents a novel species.

According to the currently accepted cut-off values of sequence similarity of the ITS and LSU markers for the delimitation of yeast species, the isolates presented above constitute eight new yeast species.

## 4. Conclusions

Our survey uncovered a remarkable diversity of yeast species associated with insects. Notably, ~42% of the yeast isolates proved to be previously unknown species, underscoring the vast potential for new discoveries in unexplored fungal populations interacting with insects globally.

Our results affirm the role of insects as vectors for the dispersal of opportunistic yeast in natural environments.

## Figures and Tables

**Table 1:** Insect collection sites in Bulgaria: site number on the map above, region, the year of collection and altitude.

Site No on the Map	Region	2008–2012 Season	Altitude
1	National Park Zlatni Pyasatsi	23–24 April/4–5 June	70–220 m
2	Eastern Rhodopes Mountains, 1.5 km E Meden Buk village	30 April	114 m
3	Eastern Rhodopes Mountains, 3 km SW Malko Gradishte village	14 July	530 m
4	Belasitsa Mountain, Samujlovo village, Klyuch village, Kamena village, Belasitsa Chalet-Vodopada Place	9 May/2–3 August	400–700 m
5	Lozenska Planina Mountain, Gabra village	24 May	800–900 m
6	Osogovska Planina Mountain, 0.5 km N Chetirchi village	25 May	440–500 m
7	Osogovska Planina Mountain, near Iliya village	28 September	600–700 m
8	Osogovska Planina Mountain, 1 km W Kyustendil City	29 September	530–600 m
9	Sofia City: Slatina District, Darvenitsa District	7–15 July/8 September	600–650 m
10	Nature park Vitosha, above Bistritsa village	19 July	600–1000 m
11	National park Rila, Rila manastery environments	28 July	1300–1400 m
12	Podgorie Region, Sandanski—Petrich valley	9 August	400–500 m
13	Struma River, Kozhuh Hill, Rupite, General Todorov village	3 July/9 August	120–130 m
14	National park Pirin, between Jane Sandanski Hut and Kamenitsa Hut	31 July–1 August	1400–1600 m
15	Thracian Lowland, Lesichovo village	6 May	300 m

## Data Availability

The new sequences generated in this study have been deposited in GenBank under the accession numbers listed in Tables S1 and S2 of the Supplementary Materials. Additionally, raw data and other relevant materials can be requested from the corresponding author upon reasonable request.

## Supplementary Materials

The following supporting information can be downloaded at https://www.mdpi.com/article/10.3390/insects15080566/s1, Table S1: List of *Ascomycetous* yeasts isolated in this study, locality and habitat, isolation hosts, and accession numbers of LSU and ITS sequences in GenBank database; Table S2: List of Basidiomycetous yeasts isolated in this study, locality and habitat, isolation hosts, and accession numbers of LSU and ITS sequences in GenBank database.
