# Supplementary material for: First DNA Barcoding Survey in Bulgaria Unveiled Huge Diversity of Yeasts in Insects
Source: Insects. 2024 Jul 26;15(8):566. doi: 10.3390/insects15080566 (PMC11354417; doi:10.3390/insects15080566)
Supplement: Supplementary file 1 [file insects-15-00566-s001.zip › insects-3056995-supplementary.pdf]

Table S1. List of *Ascomycetous* yeasts isolated in this study, locality and habitat, their isolation hosts, and accession numbers of LSU and ITS sequences in GenBank database

Strain	Yeast/Host	Locality/Habitat	GenBank Accession Number LSU/ITS
IMB-1R	***Scheffersomyces stipitis*** larva of *Cetonia aurata* (L.) (Coleoptera: Cetoniidae)	Nature park Zlatni Pyasatsi, under a rotten stump	HM627052.1/HM627161.1
D6	***Scheffersomyces stipitis*** *Dorcus parallelipipedus* (L.) (Coleoptera: Lucanidae)	Nature park Zlatni Pyasatsi, an oak forest, in a rotten stump	HM627089.1/HM627151.2
D141_1	***Scheffersomyces stipitis*** *Rhaesus serricollis* (Motsch.) (Coleoptera: Cerambycidae)	a bank of Struma River, from a plant	JQ026364.1/JQ026363.2
D161	***Scheffersomyces stipitis*** *Aegosoma scabricorne* (Scopoli) (Coleoptera: Cerambycidae	Podgorie region, from an old chestnut tree	HM627073.1/KC349926.2
D_LAR	***Scheffersomyces stipitis*** Coleoptera gen. sp., larva	Sofia city, on a tree trunk	HM627076.2/JF693330.2
D10	***Scheffersomyces insectosa*** *Euboeus tenebricosus* (Brullé) (Coleoptera: Tenebrionidae)	Nature park Zlatni Pyasatsi, an oak forest, under a rotten stump	HM627124.2/JQ026330.1
D42	***Scheffersomyces insectosa*** *Rutpela maculata* (Poda) (Coleoptera: Cerambycidae)	Nature park Zlatni Pyasatsi, on a flower	HM627102.1/JF693337.1
D42_Pir	***Scheffersomyces schehatae*** *Rutpela maculata* (Poda) (Coleoptera: Cerambycidae)	National park Pirin, from a flower	HM627122.1/JQ026334.1
D44	***Scheffersomyces insectosa*** *Stenurella bifasciata* (O. F. Müller) (Coleoptera: Cerambycidae)	Nature park Zlatni Pyasatsi, from a flower	HM627107.2/JQ026335.1
D45	***Scheffersomyces insectosa*** *Clytus rhamni* Germar (Coleoptera: Cerambycidae)	Nature park Zlatni Pyasatsi, from a flower	HM627103.1/KC118119.1
D46-2	***Scheffersomyces insectosa*** *Chlorophorus figuratus* (Scopoli) (Coleoptera: Cerambycidae)	Nature park Zlatni Pyasatsi, from a flower	HM627104.1/JQ026339.2
D52	***Scheffersomyces insectosa*** *Stictoleptura scutellata* (F.) (Coleoptera: Cerambycidae)	Belasitsa mountain, on a birch	HM627106.2/KC118120.1
D102_B	***Scheffersomyces insectosa***	National park Pirin,	HM627086.2/HM627167.2

	Zygaenidae gen. sp.<br>(Lepidoptera)	from a flower	
D306	***Scheffersomyces insectosa***<br>Coleoptera gen. sp.	Sofia city, Darvenitsa district<br>from a flower	HM627055.1/JQ026376.1
DUN1	***Scheffersomyces insecctosa***<br>Coleoptera gen. sp.	Sofia city, Darvenitsa district<br>from a flower	HM627060.2/JQ026374.1
DCHZ	***Scheffersomyces insectosa***<br>Coleoptera gen. spp.	Sofia city, Darvenitsa district<br>from a flower	JQ026378.1/JQ026379.2
D47-1	***Suhomyces atakaporum***<br>*Nalassus dryadophilus* (Mulsant)<br>(Coleoptera: Tenebrionidae)	Nature park Zlatni Pyasatsi, within<br>an oak forest, beneath the soil,<br>under a decaying stump	JQ026340.1/JQ026341.1
D47-3	***Suhomyces atakaporum***<br>*Nalassus dryadophilus* (Mulsant)<br>(Coleoptera: Tenebrionidae)	Nature park Zlatni Pyasatsi, within<br>an oak forest, beneath the soil,<br>under a decaying stump	HM627108.3/JQ026342.1
D48	***Suhomyces atakaporum***<br>*Triplax russica* (L.)<br>(Coleoptera: Erotylidae)	Nature park Zlatni Pyasatsi, within<br>an oak forest, beneath the soil<br>under a decaying stump	HM627105.2/JF693336.1
D49S	***Suhomyces atakaporum***<br>*Orchesia micans* (Panzer)<br>(Coleoptera: Melandryidae)	Nature park Zlatni Pyasatsi, within<br>an oak forest, under a decaying stump	HM627071.1/JQ026343.1
D34-1	***Debaryomyces* spp**.<br>*Lachnaia sexpunctata* (Scopoli)<br>(Coleoptera: Chrysomelidae)	Osogovska Planina mountain,<br>from a meadow	HM627066.1/JQ026331.1
D139-41	***Debaryomyces* spp.**<br>Orthoptera gen. sp.	a bank of Struma river, from<br>a plant	JQ026361.1/JQ026362.1
D1	***Schwanniomyces polymorphus***<br>*Cetonia aurata* (L.), larva<br>(Coleoptera: Cetoniidae)	Nature park Zlatni Pyasatsi,<br>under a rotten stump	HM627132.2/HM627164.1
D11	***Schwanniomyces polymrphus***<br>*Harpalus anxius* (Duftschmid)<br>**(**Coleoptera: Carabidae)	a bank of Struma River, Kozhuh<br>Hill, on a plant	HM627125.2/HM627159.1
D26	***Candida savonica***<br>Ciidae gen. sp.<br>(Coleoptera: Ciidae)	Sofia region, Gabra Village,<br>from *Trametes versicolor*<br>(Polyporaceae)	HM543265.1/KC349936.2
D61	***Candida savonica***<br>Ciidae gen. sp.<br>(Coleoptera: Ciidae)	Sofia, from *Trametes versicolor*<br>(Polyporaceae)	JQ026349.1/JQ026350.1
D89	***Candida savonica***<br>Ciidae gen. sp.<br>(Coleoptera: Ciidae)	Nature park Vitosha, birch forest,<br>from *Trametes versicolor*<br>(Polyporaceae)	HM627095.1/JQ026355.1
D96W	***Candida schatavii***<br>Orthoptera gen. sp.	National park Pirin, from a meadow	HM627114.1/JQ026358 .1

IMBCIS	***Candida boleticola***<br>*Ganoderma* sp.<br>(Ganodermataceae)	Sofia city, on a tree trunk	KC878456.1/KC878463.1
D148-4	***Metschnikowia pulcherrima***<br>Lepidoptera gen. sp.	a bank of Struma river,	HM627099.1/JQ026365.1
IMBCO2	***Metschnikowia pulcherrima***<br>*Bombus* sp.<br>(Hymenoptera: Apidae)	Nature park Vitosha, from a flower	KC878450.2/KC878460.1
D119_1	***Metschnikowia reukaufii***<br>Buprestidae gen. sp.<br>(Coleoptera)	National park Pirin, from a flower	HM627129.1/JQ026360.1
IMB43Pir	***Hypopichia rhagii***<br>*Dinoptera collaris* (L.)<br>(Coleoptera: Cerambycidae)	National park Pirin, from a flower	HM627123.1/KC810956.1
D123	***Hypopichia rhagii***<br>Buprestidae gen. sp., larva<br>(Coleoptera)	National park Pirin, under a bark in coniferous forest	HM627119.2/ KC349930.1
D53	***Saccharomyces* sp.**<br>*Mimela aurata* (Fabricius)<br>(Coleoptera: Rutelidae)	Belasitsa mountain, on a *Platanus orientalis*	HM627121.1/JQ026346.1
IMB50-2	***Lachancea kluyveri***<br>*Purpuricenus budensis* (Götz)<br>(Coleoptera: Cerambycidae)	Kozuh hills, village General Todorov from a meadow	HM627120.2/HM627158.1
DB2	***Hanseniaspora uvarum***<br>Coleoptera gen. sp.	Sofia city, from a plant	HM627056.2/JQ026377.1
IMBT3	***Hanseniaspora uvarum***<br>*Forficula auricularia* (L.)<br>(Insecta: Dermaptera)	Sofia city, from a vine tree	KC349933.1/KC349934.1
IMB37-4	***Starmerella bombicola***<br>*Valgus hemipterus* (L.)<br>(Coleoptera: Scarabaeidae)	Osogovska Planina mountain, from a meadow	KC878455.1/ HM627155.1
IMBC45	***Starmerella bombi***<br>*Bombus* sp.<br>(Hymenoptera: Apidae)	Nature park Vitosha, from a flower	KC878449.1/KC878459.1
D77_3	***Nakaseomyces glabratus***<br>*Oxythyrea funesta* (Poda)<br>(Coleoptera: Cetoniidae)	Nature park Vitosha, Bistritsa village birch forest, under a stone	HM627130.1/JQ026352.1
D171	***Meyerozyma guilliermondii***<br>*Bitoma crenata* (Fabricius)<br>(Coleoptera: Colydiidae)	Podgorie region, from *Ganoderma applanatum* (Ganodermataceae)	JQ026368.1/JQ026369.1
IMB205_2	***Pichia norvegensis***<br>*Cybister lateralimarginalis* (De Geer)<br>(Coleoptera: Dytiscidae)	Osogovska Planina mountain, under a stone	HM627085.1/JQ026372.1

D141-4	***Candida maltosa*** *Rhaesus serricollis* (Motsch.) (Coleoptera: Cerambycidae)	a bank of the Struma River, from a plant	HM627098.1/KC349927.1
IMBR1R	***Kluyveromyces dobzhanskii*** *Cetonia aurata* (L.), larva (Coleoptera: Cetoniidae)	East Rhodopes, from a flower	HM627117.1/ KC810947.1
D50-3	***Pichia membranifaciens*** *Purpuricenus budensis* (Götz) (Coleoptera: Cerambycidae)	Kozuh hills, General Todorov village from a meadow	JQ026344.1/JQ026345.1
D88_B	***Exophiala sideris*** (Collembola: Sminthuridae gen. sp.)	Nature park Vitosha, from a fungus	HM627072.1/KC315801.1
D30k	***Teunomyces kruisii*** Coleoptera gen. sp.	Sofia city, from a plant	HM627126.2/JQ026373.1
D_MR	***Yarrowia lipolitica*** Formicidae gen. sp. (Insecta: Hymenoptera)	Lesichovo village, on the ground	HM627078.2/HM627146.2
IMB60-4	***Torulaspora delbrueckii*** unidentified wasp (Hymenoptera: Apocrita)	Sofia city, on the ground	KC878453.1/ KC878462.1
D124	***Ogataea saltuana*** [85] Scolitinae gen. sp. (Coleoptera: Curculionidae)	National park Pirin, under a bark in coniferous forest	HM627128.1/JF923536.1
NBIMCC* 8926 CBS* 12431	***Priceomyces vitoshaensis*** [86] *Pterostichus melas* (Creutzer) (Coleoptera: Carabidae)	Nature park Zlatni Pyasatsi an oak forest, under a tree trunk	KC810946.1/KC810955.1
NBIMCC 8927 CBS 12457	***Priceomyces vitoshaensis*** [86] *Pterostichus melas* (Creutzer) (Coleoptera: Carabidae)	Nature park Vitosha, a birch forest, under a stone	HM627053.2/HM627157.2
NBIMCC 8862 CBS 12463	***Candida cetonia*** [37] *Oxythyrea funesta* (Poda) (Coleoptera: Cetoniidae)	Nature park Vitosha, Bistritsa village, birch forest, under a stone	KC118128.3/KC118129.3
NBIMCC 8863 CBS 12442	***Candida cetonia*** [37] *Cetonia aurata* (L.) (Coleoptera: Cetoniidae)	East Rhodopes, from a flower	KC810948.1/KC315799.1
NBIMCC 8864 CBS 12564	***Nematodospora valgi*** [37] *Valgus hemipterus* (L.) (Coleoptera: Scarabaeidae)	Osogovska Planina mountain Chetirchi village, from a meadow	KC315802.1/ KM386995.1
NBIMCC 8865 CBS12565	***Nematodospora valgi*** [37] *Valgus hemipterus* (L.) (Coleoptera: Scarabaeidae)	Nature park Zlatni Pyasatsi from a meadow	HM627058.1/JF693335.1
NBIMCC 8866 CBS12563	***Nematodospora valgi*** [37] *Valgus hemipterus* (L.) (Coleoptera: Scarabaeidae)	Osogovska Planina mountain, Chetirchi village, from a meadow	HM627135.2/HM627165.2

NBIMCC 8867<br>CBS 12561	***Nematodospora valgi*** [37]<br>*Valgus hemipterus* (L.)<br>(Coleoptera: Scarabaeidae)	Osogovska Planina mountain<br>Chetirchi village, from a meadow	HM627135.2/HM627165.2
NBIMCC 8868<br>CBS12562	***Nematodospora valgi*** [37]<br>*Valgus hemipterus* (L.)<br>(Coleoptera: Scarabaei	Osogovska Planina mountain,<br>Chetirchi village, from a meadow	HM627112.1/ KM386993.1
NBIMCC 8888<br>CBS 12462	***Yarrowia parophonii*** [87]<br>Coleoptera gen. sp.<br>(Coleoptera: Carabidae)	Lesichovo village, under a<br>tree bark	KC810944.1/KC810951.1
NBIMCC 8889<br>CBS12427	*Yarrowia parophonii* [93]<br>*Parophonus hirsutulus* (Dejean)	a bank of the Struma river, from<br>a plant	JQ026370.2/JQ026371.2
NBIMCC 8900<br>CBS 12468	***Yarrowia parophonii*** [87]<br>Coleoptera gen. sp.	Lesichovo village, under a<br>a tree bark	KY457253.1/KY457248.1
NBIMCC 8901<br>CBS 12441	***Yarrowia parophonii*** [87]<br>Coleoptera gen. sp.	Lesichovo village, under a<br>tree bark	KY457257.1/ KY457252.1
NBIMCC 8902<br>CBS 12466	***Yarrowia parophonii*** [87]<br>Coleoptera gen. sp.	Lesichovo village, under a<br>tree bark	KY457254.1/KY457249.1
NBIMCC 8903<br>CBS 12465	***Yarrowia parophonii*** [87]<br>Coleoptera gen. sp.	Lesichovo village, under a<br>tree bark	KY457256.1/ KY457251.1
NBIMCC 9804<br>CBS 12471	***Yarrowia parophonii*** [87]<br>Coleoptera gen. sp.	Lesichovo village, under a<br>tree bark	KY457255.1/ KY457250.1
NBIMCC 9028<br>CBS12566	***Kazachstania molopis*** [88]<br>*Molops piceus* (Panzer)<br>(Coleoptera: Carabidae)	Nature park Zlatni Pyasatsi<br>birch forest, under a stone	HM627092.1/HM627145.1
NBIMCC 9029<br>CBS 12448	***Kazachstania molopis*** [88]<br>*Molops piceus* (Panzer)<br>(Coleoptera: Carabidae)	Nature park Zlatni Pyasatsi<br>a birch forest, under a stone	KC878454.1/KC118123.1
NBIMCC 8992<br>CBS12424	***Kazachstania chrysolinae*** [39]<br>*Chrysolina polita* (L.)<br>(Coleoptera: Chrysomelidae)	Podgorie region, from a plant	HM627101.1/JF693338.1
NBIMCC 8993<br>CBS 12446	***Kazachstania chrysolinae*** [39]<br>*Chrysolina polita* (L.)<br>(Coleoptera: Chrysomelidae)	Podgorie region, from a plant	KC118126.1/KC118127.1
NBIMCC 8992<br>CBS12443	***Suhomyces rilaensis*** [89]<br>*Bolitophagus interruptus* Illiger<br>(Coleoptera: Tenebrionidae)	Podgorie region, from *Ganoderma applanatum* (Ganodermataceae)	HM627061.2/HM627147.2
NBIMCC 8930<br>CBS12453	***Suhomyces rilaensis*** [89]<br>*Bolitophagus reticulatus* (L.)<br>(Coleoptera: Tenebrionidae)	Rila monastery area,<br>from *Fomes fomentarius,* on a<br>birch tree	HM627113.1/HM627148.2
NBIMCC 8931<br>CBS12460	***Suhomyces rilaensis*** [89]<br>Orthoptera gen. sp.	Rila monastery area,<br>from a meadow	HM627116.2/HM627150.1

CBS 5659	***Starmerela xylocopis*** [90]<br>*Xylocopa caffra* South Africa<br>(Hymenoptera: Apidae)	from a larval feed	AY521569.1/KF181968.1
CBS 6396	***Starmerela xylocopis*** [90]<br>*Xylocopa scioensis*<br>(Hymenoptera: Apidae)	from the larval pabulum<br>South Africa	AY521570.1/KF181965.1
DZ1_1<br>CBS 12458	***Wickerhamiella* spp.**<br>*Bolitophagus interruptus* Illiger<br>(Coleoptera: Tenebrionidae)	Podgorie region, from *Ganoderma*<br>spp. (Ganodermataceae)	HM627062.2/JF693332.3
DZ2_1<br>CBS 12464	***Wickerhamiella* spp.**<br>*Bolitophagus interruptus* Illiger<br>(Coleoptera: Tenebrionidae)	Podgorie region, from *Ganoderma*<br>spp. (Ganodermataceae)	HM627057.2/JF693333.2
D300<br>CBS 12467	***Wickerhamiella* spp.**<br>*Bolitophagus reticulatus* (L.)<br>(Coleoptera: Tenebrionidae)<br>*Betula* spp. (Betulaceae)	Rila monastery area,<br>from a *Fomes* spp., on a	HM627091.2/HM627168,2
IMB-P1<br>NBIMCC 8921<br>*Salix* spp. (Salicaceae)	***Suhomyces* spp.**<br>Coleoptera gen. sp.	Sofia city, Darvenitsa district<br>from a *Laetiporus sulphureus* onCBS 12737	KC 349940.1/ JX656733.1
IMB-PP2<br>NBIMCC 8923<br>CBS 12433	***Suhomyces* spp.**<br>*Porcellio scaber* Latreille<br>(Isopoda: Porcellionidae)	Sofia city, Darvenitsa district<br>from a *Laetiporus sulphureus.* on a<br>*Salix* spp. (Salicaceae)	JX656731.1/ JX656732.1
MB-P21<br>NBIMCC 8910<br>CBS 12739	***Suhomyces* spp.**<br>*Forficula* sp.<br>(Dermaptera: Forficulidae)	Sofia city, Darvenitsa district<br>from a *Laetiporu sulphureus* on<br>*Salix* spp. (Salicaceae)	JX656736.2/JX656737.3
IMB-P43<br>NBIMCC 8928<br>CBS 12740	***Suhomyces* spp.**<br>Coleoptera gen. sp.	Sofia city, Darvenitsa district,<br>from a *Laetiporus sulphureus*<br>on a *Salix* spp. (Salicaceae)	JX656740.1/ JX656741.1
IMB-P5<br>NBIMCC 8911<br>CBS 12741	***Suhomyces* spp.**<br>*Forficula auricularia* (L.)<br>(Dermaptera: Forficulidae)	Sofia city, Darvenitsa district,<br>from a *Laetiporus sulphureus*<br>on a *Salix* spp. (Salicaceae)	JX656736.1/JX656737.1
IMB-P72<br>NBIMCC 8912<br>CBS 12742	***Suhomyces* spp.**<br>*Scaphidium quadrimaculatum*<br>Olivier (Coleoptera:<br>Staphylinidae)	Sofia city, Darvenitsa district,<br>from a *Laetiporus sulphureus* on<br>a *Salix* spp. tree	JX656742.1/ JX656743,1
IMB-PG2<br>NBIMCC 8914<br>CBS 12745	***Suhomyces* spp.**<br>Insecta, larva	Sofia city, Darvenitsa district,<br>from a *Laetiporus sulphureus* on<br>a *Salix* spp. (Salicaceae)	JX656734.2/ JX656735.2
IMB-YP<br>NBIMCC 8922<br>CBS 12425	***Suhomyces* spp.**<br>*Laetiporus sulphureus*<br>(Polyporales)	Sofia city, Darvenitsa district,<br>from a *Salix* sp. (Salicaceae)	JX656725.1/JX656726.1
IMB-YP8<br>NBIMCC 8913	***Suhomyces* spp.**<br>*Laetiporus sulphureus*	Sofia city, Iztok district, on a<br>*Acacia* sp. (Fabaceae)	KC810943.2/KC810950.1

CBS 12743	(Polyporales)		
IMB-PX1<br>NBIMCC 8925<br>CBS 12744	***Suhomyces* spp.**<br>Coleoptera gen. sp.	Sofia city, Darvenitsa district, from *Laetiporus sulphureus* on a *Salix* sp. (Salicaceae)	JX656728.2JX656727.2
D15<br>NBIMCC 8878<br>CBS 12444	***Diutina* spp.**<br>*Hister quadrimaculatus* L. (Coleoptera: Histeridae)	East Rhodopes, an oak forest near Biala river	HM627111.3/HM627160.1
D24<br>NBIMCC 8879<br>CBS 12428	***Diutina* spp.**<br>*Trichodes crabroniformis* (F.) (Coleoptera: Cleridae)	Belasitsa mountain, from a a flower	HM627090.2/HM627153.NBIMCC
D91W<br>NBIMCC 8823<br>CBS 12429	***Starmerella* spp.**<br>*Carabus violaceus azurensis* Dejean (Coleoptera: Carabidae)	National park Pirin, coniferous forest, under a stone	JQ026336.1/JQ026357.1
D91WR<br>NBIMCC 8924<br>CBS 12430	***Starmerella* spp.**<br>*Carabus violaceus azurensis* Dejean (Coleoptera: Carabidae)	National park Pirin, coniferous forest, under a stone	KF850165.1/KF856945.1
DZ1_2<br>CBS 12434	***Blastobotrys* spp.**<br>*Bolitophagus interruptus* Illiger (Coleoptera: Tenebrionidae)	Podgorie region, from *Ganoderma* spp. (Ganodermataceae)	HM627063.1/KC878464.1
DZ2_2<br>CBS 12455	***Blastobotrys* spp.**<br>*Bolitophagus interruptus* Illiger (Coleoptera: Tenebrionidae)	Podgorie region, from *Ganoderma* spp. (Ganodermataceae)	HM627059.1/JQ026375,1

Table S2. List of Basidiomycetous yeasts isolated in this study, locality and habitat, their isolation hosts, and accession numbers of LSU and ITS sequences in GenBank database

Strain	Yeast/Host	Locality/Habitat	GenBank Accession Number LSU/ITS
D33	***Filobasidium stepposum***<br>*Galeruca tanaceti* (L.)<br>(Coleoptera: Chrysomelidae)	Osogovska Planina mountain, Chetirchi village, from a meadow	HM627067.1/JQ026332.1
D-54	***Trichosporon* spp.**<br>*Anomala osmanlis*<br>(C.É. Blanchard)<br>(Coleoptera: Rutelidae)	Kozhuh Hills, from a meadow	JQ026348.1/JQ026347.1
D55	***Trichosporon* spp.**<br>*Anoxia orientalis* (Krynicki)<br>(Coleoptera: Melolonthidae)	a bank of Struma river, from a meadow	HM627118.1/HM627169.1
D59-1	***Trichosporon* spp.**<br>*Cheironitis furcifer* (P. Rossi)<br>(Coleoptera: Scarabaeidae)	Kozhuh Hills, from a meadow	HM627054.1/HM627162.2
D60-1	***Trichosporon* spp.**<br>*Bembidion dalmatinum* (Dejean)<br>(Coleoptera: Carabidae)	Belasitsa mountain, *Platanus orientalis* forest	HM627134/HM627163.2
D83_ST	***Apiotrichum lignicola***<br>*Anoplotrupes stercorosus* (Scriba)	Nature park Vitosha, birch forest	HM627093.1/JQ026354.1

	(Coleoptera: Geotrupidae)		
D84	***Apiotrichum lignicola***<br>*Jekelius punctulatus* (Jekel)<br>(Coleoptera: Geotrupidae)	Nature park Vitosha, birch forest, from the ground	HM627094/KC349938.2
CBS 12423	***Cystobasidium psychroaquaticum*** [95]<br>(Collembola: Sminthuridae gen.sp.)	forest, from a fungus	JN572894.1/JN572895.1
D116	***Vanrijia albida***<br>*Sphaerosoma* sp.<br>(Coleoptera: Alexiidae)	National park Pirin, from a fungus	HM627068.1/JQ026359.1
IMB139_2	***Rhodotorulla mucilaginosa***<br>Orthoptera gen. sp.	a bank of the Struma river, from a plant	KC349929.1/KC349928.1
D139_3	***Trichosporon coremiiforme***<br>Orthoptera gen. sp.	a bank of the Struma river, from a plant	HM627096.2/ KC954608.1
IMB162_1	***Rhodosporidium kratochvilovae***<br>*Aegosoma scabricorne* (Scopoli)<br>(Coleoptera: Cerambycidae)	Podgorie region, an old mesophilous forest	JQ026366.1/JQ026367,1
D162_2<br>CBS 12426	***Saitozyma* spp.**<br>*Melandrydae* gen. sp.<br>(Coleoptera)	Podgorie region, an old mesophilous	HM627081.1/JF693331,1
IMB169	***Filobasidiella oeirensis***<br>*Uleiota planata* (L.)<br>(Coleoptera: Silvanidae)	Belasitsa mountain, from a polypore fungus	HM627110.2/KC118116.1
D179	***Filobasidiella oeirensis***<br>Buprestidae gen. sp.<br>(Coleoptera)	Rupite village, from a plant	HM627100.1/KC118117.1
D187_2	***Filobasidiella wieringae***<br>*Chlorophorus varius*<br>(O. F. Müller)<br>(Coleoptera: Cerambycidae)	Rupite village, from a plant	HM627074.3/HM627149.2
D206	***Apiotrichum humicola***<br>Staphylinidae gen. sp.<br>(Coleoptera)	Osogovska Planina mountain, from a polypore fungus	HM627084/KC118118.1

*NBIMCC; National bank for Industrial Microorganisms and Cell Cultures, Sofia Bulgaria

**CBS; Culture collection of the Westerdijk Fungal Biodiversit Institute, Utrecht, The Netherlands
